# A murine malaria protocol for characterizing transmission blocking benefits of antimalarial drug combinations

**Published:** 2020-04-01

**Authors:** Yehenew A. Ebstie, Alain R. Tenoh Guedoung, Annette Habluetzel

**Affiliations:** 1 School of Pharmacy, University of Camerino, Camerino, Italy; 2 Centro Interuniversitario di Ricerca sulla Malaria/Italian Malaria Network, University of Milan, Milan, Italy

## Abstract

**Background:**

Current efforts towards malaria elimination include the discovery of new transmission blocking (TB) drugs and identification of compounds suitable to replace primaquine, recommended as transmission blocking post treatment after artemisinin combination therapy (ACT). High through put screening of compound libraries has allowed to identify numerous compounds active *in vitro* against gametocytes and insect early sporogonic stages, but few studies have been performed to characterize TB compounds *in vivo*. Here we propose a double TB drug Direct Feeding Assay (2TB-DFA), suitable to assess the combined effects of TB compounds.

**Materials and methods:**

*Plasmodium berghei* GFPcon (*PbGFPcon*), BALB/c mice and *Anopheles stephensi* mosquitoes were used. Artemisinin (ART) and artesunate (AS) served as examples of artemisinins, NeemAzal® (NA), as a known TB-product with sporontocidal activity. DFA experiments were performed to assess the appropriate time point of administration before mosquito feeding and estimate suitable sub-optimal doses of the three compounds that allow combination effects to be appreciated.

**Results:**

Suboptimal dosages, that reduce about 50% of oocyst development, were recorded with ART in the range of 16-30 mg/ kg, AS 14-28 mg/kg and NA 31-38mg/kg. Ten hours before mosquito feeding (corresponding to 3.5 days after mouse infection) was determined as a suitable time point for mouse treatment with ART and AS and 1 hour for post-treatment with NA. ART given at 35 mg/kg in combination with NA at 40 mg/kg reduced oocyst density by 94% and prevalence of infection by 59%. Similarly, the combination of ART at 25 mg/kg plus NA at 35 mg/kg decreased oocyst density by 95% and prevalence of infection by 34%. In the 2TB-DFA, conducted with AS (20 mg/kg) and NA (35 mg/kg) the combination treatment reduced oocyst density by 71% and did not affect prevalence of infection. Applying ‘Highest Single Agent’ analysis and considering as readout oocyst density and prevalence of infection, cooperative effects of the combination treatments, compared with the single compound treatments emerged.

**Conclusion:**

This study suggests the 2TB-DFA to be suitable for the profiling of new TB candidates that could substitute primaquine as a post-treatment to ACT courses.

## 1 Introduction

Global, sustained efforts have allowed rolling back the malaria burden substantially in the first fifteen years of the 20^th^ century. Globally, between 2000 and 2015, malaria incidence fell by 37% and malaria mortality by 60% [1]. Since a few years, however, trend curves of malaria case incidence and death rates are flattening and the danger of losing the hard-won achievements is in front of our eyes [2]. The plight calls for substantial funding to endemic countries for more efficient implementation of available tools. This regards in particular artemisinin combination therapy (ACT), the current first line treatment for uncomplicated malaria and insecticide-treated bednets protecting populations at risk from infectious mosquito bites. Given the specter of *Plasmodium falciparum* parasites and *Anopheles* vectors developing resistance to the currently used drugs and insecticides, respectively [2], multiple, innovate tools are required for maintaining malaria control achievements and reach the ambitious objective of disease eradication.

ACTs, though very effective in killing asexual blood stages and curing of malaria patients, are not able to completely clear mature gametocytes from the bloodstream, so that ACT-treated individuals remain – albeit to a minor extent - infective to bloodfeeding mosquitoes for about one to three weeks after treatment [3]. Primaquine, given as post-ACT treatment can remove mature gametocytes efficiently but has raised safety concerns in glucose-6-phosphate dehydrogenase deficient (G6PD) patients when given at 0.75 mg base/kg [4]. Based on a review of the evidence of safety and effectiveness of primaquine as a *P. falciparum* gametocytocide, the current WHO recommendation indicates to use the drug at 0.25 mg base/kg, which is effective in blocking transmission and unlikely to cause serious toxicity in subjects with any of the G6PD variants [4].

The need of new antimalarial drugs and more effective combination treatments, able to cure individuals and concurrently reduce transmission at population level is widely recognized [2]. Endorsing the concept, the Medicines for Malaria Venture (MMV), a product development partnership in the field of antimalarial drug research and development, proposes to orient research and development efforts towards two Target Product Profiles (TPP) and six different Target Candidate Profiles (TCP) [5]. Among them, TCP 5 and TCP 6, focus on transmission-blocking drugs and TPP 1 defines drugs (combinations) that are effective against resistant strains, can cure clinical malaria, stop transmission and prevent relapse in a single encounter [6,7].

In response to the outlined drug discovery requirements, sustained efforts to improve *in vitro* culture of the different life cycle stages combined with the development of robotic automation and high content imaging methodologies have allowed the screening of huge numbers of compounds over the last decade [8]. Asexual blood stage screens of more than two million compounds from various libraries have identified thousands of hits. The more recently developed high through put screening platforms focus on drug activity against transmissible stages, i.e. to gametocytes (stage specific and sex specific), gametes (sex specific) and ookinetes have greatly facilitated the discovery of compounds with dual- or multi-stage activity [8].

A few years ago, MMV distilled the more than 25000 compounds that kill asexual blood stages *in vitro* to 400 representative compounds, called the Malaria Box, and made this freely available to more than 200 interested research laboratories [9]. Data from 24 phenotypic screens confirmed activity on asexual blood stages. Out of the 400 compounds 257 revealed activity against young or mature gametocytes in at least 3 of the 33 studies included in the Van Voorhis meta-analysis [9]. In addition, more than 25% of the compounds (117/400) tested positive against early sporogonic stages when tested at 10 μM in the ookinete development assay [9,10]. Interestingly, several compounds, such as MMV665878, MMV66594, MMV0004481, have shown impact on various proliferative and reproductive stages within the *Plasmodium* life cycle, namely on asexual blood stages, young and mature gametocytes but also on insect early sporogonic development, i.e. on processes spanning from gamete formation to fecundation, zygote formation and ookinete maturation [9]. Such a ‘large spectrum’ activity pattern raises the question whether multiple compounds display a single mode of action targeted to a particular enzyme or receptor in a biological process common to different stages or whether compound effects on the various stages are exerted through diverse mode of actions. Target identification of hit compounds may elucidate these questions.

Drug discovery and development relies on a panel of robust *in vitro* methodologies for assessing most of the druggability features considered by preclinical evaluation. However, *in vivo* studies, involving host organisms as a whole, are still playing important roles: the classical Peters’s 4 days test [11] that employs rodent *Plasmodium* species such as *Plasmodium berghei* and *P. yoelii* in various mouse strains, allows to elucidate compounds’ bioavailability and estimate the curative potential of test compounds; the development of humanised mouse models permits to explore drug efficacy against *P. falciparum* liver and blood stages in vertebrate hosts [12] and the Direct Membrane Feeding Assay (DMFA) allows to estimate compound activity on transmissible stages, measuring infection of mosquito hosts after administration of compounds to female mosquitoes via membrane feeding of *P. falciparum* gametocytemic blood [13,14].

In a long standing collaboration with academic research groups making part of the CIRM (Centro Inter-universitario di Ricerca sulla Malaria) – Italian Malaria Network, our lab has examined numbers of compounds in the Peters’ four days test, provided by chemistry and phenotypic screening partners of the network [15,16]. Given the importance of identifying new transmission blocking drugs, we established the ookinete development assay [10] and have subsequently characterised dozens of compounds and plant extracts for activity on early sporogonic stages [17,18]. Active TB compounds and plant extracts have been validated *in vivo* with the Direct Feeding Assay (DFA) using a *P. berghei* strain (PbGFPcon) that expresses a green fluorescent protein in all life cycle stages, BALB/c mice and *Anopheles stephensi* mosquitoes. Among the substances that were found to be active, both *in vitro* and *in vivo*, compounds and products from the neem tree, *Azadirachta indica*, have yielded interesting results. Azadirachtin A and deacetylnimbin isolated from neem fruits reduce 50% of early sporogonic development at 11-14 μM [19] and 6-25 μM [20], respectively. NeemAzal®, a commercial product with an azadirachtin A content of about 50%, was found to exhibit a slightly stronger activity against early sporogonic stages (IC_50_ 6 – 8 μM) than pure azadirachtin. *In vivo*, NeemAzal® blocks mosquito infection when given at an azadirachtin A dose of 50 mg/kg to gametocytemic mice 1 h before exposure of mice to mosquitoes [21].

Based on the need for new combination treatments (MMV: TPP1) that effectively cure patients but are also able to impact on transmission, here we propose a TB *in vivo* protocol suitable for characterizing TB effects of compounds given in combination in the *P. berghei* murine model. The protocol was set up to address the urgent need of identifying appropriate post-treatments to be employed after ACT administration. Thus, bearing in mind the limited gametocytocidal activity of artemisinin derivatives and the safety concerns regarding the TB effective primaquine, the protocol was designed to evaluate additional benefits of TB candidates given to mice pre-treated with artemisinin derivatives at sub-optimal dosages, simulating persistent gametocyte circulation in individuals after ACT treatment. Artemisinin and artesunate were used as examples of ACT artemisinin derivatives and NeemAzal® as a known transmission blocking product to explore the performance of the combination protocol.

## 2 Materials and methods

### 2.1 Double-TB-Drug Direct Feeding Assay (2TB-DFA)

The protocol design is based on the administration of two TB compounds (one gametocytocidal and one sporontocidal) to gametocytemic mice at sub-optimal dosages reducing - when given alone - oocyst abundance in mosquitoes by about 50%. This allows to appreciate, an increased TB drug impact on mosquito infection when drugs are given in combination compared to the single drug effects. Thus, at first, a series of experiments was conducted with the *P. berghei* Direct Feeding Assay (DFA) and the *in vitro* ookinete development assay (ODA) to characterise transmission blocking effects of artemisinin (ART) and artesunate (AS), as known gametocytocidal drugs drugs, and of NeemAzal® (NA) as sporontocidal product. Then, dose range experiments were performed in the DFA to estimate IC_50_ ranges of the three experimental agents and finally, combination effects emerging in the double-TB-drug feeding assay (2TB-DFA) are illustrated, comparing single and double drug administration of ART with NA and AS with NA.

### 2.2 *P. berghei* model

In this study, 3-4 weeks old female BALB/c mice (weighing 20 ± 2g) were used. Animals were reared and maintained in the animal breeding facilities of the University of Camerino. Experimental protocols that involved mice have been reviewed and approved by the Italian Legislative Decree on the ‘Use and protection of laboratory animals’ (D.Lgs.116 of 10/27/ 92).

Genetically modified *Plasmodium berghei* ANKA strains (chloroquine sensitive), which express a constitutive green fluorescent protein throughout the parasite life cycle (PbGFPcon) or specifically during zygote to ookinete development (PbCTRPp.GFP) [22], were used. Parasite strains have been kindly provided by Prof. R.E. Sinden (Imperial College, London) and were maintained following standard procedures [23]. Briefly, PbGFPcon and PbCTRPp.GFP infected blood was stored in liquid nitrogen (-70°C) with glycerol as a cryo-preservative. At occurrence, capillary blood was unfrozen, diluted in PBS and intraperitoneally (i.p.) inoculated in mice. Parasite propagation was sustained through acyclic passage from infected to healthy mice through i.p. administration of parasitized red blood cells. Cyclic passages, from mice to mosquitoes and from mosquitoes to mice were routinely performed every three to four months to preserve parasite infectivity to mosquitoes. Mosquitoes infected with *P. berghei* were kept at 19 ± 1°C for the whole duration of the sporogonic cycle.

*Anopheles stephensi* mosquitoes were used as experimental vectors. Colonies were maintained at 30°C and 80-90% relative humidity with a photoperiodicity of 12hrs L:D in the insectary of the University of Camerino. Mosquitoes were reared according to standard practices [23] as described previously [21]. Adults were maintained on 8% sugar solution and females membrane blood fed for egg production. Larvae were fed with ground laboratory mouse pellets.

### 2.3 Compounds

Artemisinin, artesunate and dihydroartemisinin were purchased from Sigma**-**Aldrich (China) with purities of 98%. NeemAzal® technical grade (Trifolio-M GmbH, Lahnau, Germany) was provided by the company. According to the producer, the standardised seed kernel extract from *Azadirachta indica* contains limonoids at a concentration of 57.6%. Azadirachtin A is the most abundantly present limonoid (34%) followed by other azadirachtins B to K (16%), salanins (4%) and nimbins (2%). According to analysis performed by Orazio Taglialatela Scafati in previous NeemAzal® studies performed by our group, Azadirachtin A content reaches about 50% of the total extract [19]. In this study NA dosages were calculated based on the azadirachtin A content determined by our group.

To conduct the *in vitro* ookinete development assay (ODA) experiments, compounds were dissolved in DMSO (AppliChem, Germany) and further diluted in ookinete medium to obtain the desired test concentrations. To perform the *in-vivo* transmission blocking experiments, artemisinin was dissolved in distilled water containing 10% DMSO and 10% Tween80 (Sigma-Aldrich, USA) and diluted in normal saline (0.85% NaCl supplied by Baker, Holland) to obtain the desired mouse treatment doses. Artesunate was dissolved in 5% sodium bicarbonate (Baker, Holland) and diluted in PBS (NaCl, KCl, KH_2_PO_4_ and Na_2_HPO_4_, were purchased from Baker, Holland). NeemAzal® was dissolved in absolute ethanol (CARLO EBRO, France) and diluted in PBS (pH 6.5) with 10% DMSO and 7.5% Tween80.

### 2.4 *In vitro* ookinete development assay

The *P. berghei* ODA was employed as described by Delves *et al.* (2012) with minor modifications [20]. Briefly, two mice were infected with PbCTRPp.GFP infected RBCs (iRBCs) from capillaries. The same day, six mice were treated with phenylhydrazine (Sigma-Aldrich, Austria) at 120 mg/kg, to stimulate erythropoiesis. Four days later, the phenylhydrazine pre-treated mice were inoculated i.p. 10^7^ iRBCs using the blood from one of the capillary infected mice (~5% parasitemia). At day 4 after mouse infection, gametocytemia was determined on Giemsa thin smears and maturity of microgametocytes, i.e. their capacity to form gametes was checked by the exflagellation assay [24]. Briefly, 5 μl of blood was taken from the tail tip of the mice and diluted (~1:30) with exflagellation medium (RPMI 1640 (Sigma-Aldrich, USA) containing 25mM HEPES, 25mM sodium bicarbonate, 50 mg/L hypoxanthine, 100μM xanthurenic acid (Sigma-Aldrich, USA), pH 8.3). Samples of diluted blood (7 μl) were then placed in hand-made glass slide chambers [19] and incubated for 20 min at 19°C. Numbers of flagellated gamete extruding microgametocytes, visible as vibrating ‘exflagellation centers’ were counted under the microscope (400×). Mice showing more than 3 exflagellation centers per 1000 RBCs and the presence of female and male gametocytes on thin smears were selected as blood donors for the ODA.

Stock solutions of artemisinin derivatives and NA were prepared at 10 mg/ml in DMSO and diluted in ookinete medium i.e. exflagellation medium adjusted to pH 7.4 and supplemented with 20% heat-inactivated fetal bovine serum (Gibco, South America), 10000 IU/ml of penicillin and 10000 μg/ml of streptomycin (Sigma-Aldrich, USA). Prediluted compounds were dispensed to the wells of 96 wells micro-plates (Falcon, USA) to obtain the desired final concentration in a total volume of 100 μl, at a final DMSO concentration of not more than 0.2% (0.2% DMSO in ookinete medium served as negative control). Infected blood collected from donor mice was added to the wells at a 1:20 dilution (corresponding to a hematocrit of 1–2%) and mixed swiftly. The plate was quickly transferred to a 19°C chamber and incubated for 22–24hr. Subsequently, each well content was mixed thoroughly (to disintegrate aggregations of ookinetes) and diluted 1:50 in PBS (pH 7.4) in a new plate to obtain a cell monolayer. After sedimentation of ESS and blood, GFP -expressing zygotes and ookinetes were counted using a fluorescence microscope (FITC fluorescent filter, 400 × magnification). All samples were tested in triplicate wells using blood from at least 2 mice on different plates.

### 2.5 Direct Feeding Assay (DFA)

Initially, four mice were infected with PbGFPcon using infected blood from capillaries stored in liquid nitrogen. Four to five days later, mice with parasitemia of about 5-7% were used to infect experimental mice with a standardized number of 10^6^ PbGFPcon infected RBCs per mouse. Female BALB/c mice weighing 18-22 g were used. On day 3 after infection, thin blood films were prepared from the tail tips of mice and parasitemia determined. Mice with a parasitemia in the range of 2-5% and presence of male and female gametocytes were selected for the DFA experiments on the following day. Three mice were allocated to each treatment and control group.

On day four after infection, compounds were administrated i.p. to the selected mice 1h (if not specified differently) before mosquito infection. Mice were then anaesthetised with xylazine: acepromazine and placed for 30 to 45 minutes over cages each containing about 50 female *An. stephensi* mosquitoes (3-5 days old) for blood feeding. Three mouse/mosquito cage replicates were prepared for each treatment group. *P. berghei* mosquito infection was performed in a climate chamber at 19 ± 1°C and 70-80% relative humidity. Unfed mosquitoes were removed 24 h after the blood meal and fed females provided with 8% sugar solution supplemented with 0.05% para-amino benzoic acid (PABA; Sigma-Aldrich, USA) to support oocyst development [23].

On day six (seven) after mosquito infection, 30 females were dissected per mosquito cage (3 x 30 = 90 per treatment group) and mid guts examined to assess the prevalence and density of oocysts using the fluorescence microscope (400×).

### 2.6 Statistical analysis

Data were entered in Excel 2007 and analysed with GraphPad Prism 5. P-values below 0.05 were considered statistically significant. Normal distributed data (ESS numbers and their % inhibition) were expressed as arithmetic means of well and plate replicate counts and 95% confidence intervals (CI_95%_) calculated. Oocyst densities were expressed as geometric means of 30 midgut counts per mosquito cage ± CI_95%_ and arithmetic means calculated from the 3 replicate means. Student's *t-*test was utilised to compare means of independent samples (geometric means) and Fisher's exact test was used to compare categorical data (prevalence and percentage reductions). Dose-range experiments were conducted with ART, AS and NA to estimate the compounds’ TB activity on oocyst densities in the DFA. Nonlinear regression using log transformed dose data was applied for the calculation of IC_50_ and CI_95_ values. Linear regression was used to analyse correlation of drug dose and oocyst density and the models goodness-of-fit verified by calculating the parameter R-Squared.

The ‘Highest Single Agent’ approach has been adopted to assess whether the effect of the combination is greater than the effects produced by the individual compounds [25,26]. The Combination Index was calculated as CI= max (E_A_, E_B_) / E_AB_, where E_A_ refers to the effect of artemisinin or artesunate, E_B_ to that of NeemAzal and E_AB_ to that of the combination. The significance of a positive effect was evaluated by comparing the effect of the combination to that of the highest single agent (student’s *t* test).

## 3 Results

### 3.1 Effects of artemisinins on *P. berghei* early sporogonic development *in vitro*

To answer whether artemisinins may have an effect on insect early sporogonic development primary screening was performed at 75μM (supra-pharmacological concentration). Screening of ART, DHA and AS at this concentration in the *in vitro* ookinete development assay showed ART to be inactive and minor effects were observed with DHA and AS on *P. berghei* early sporogonic development (Table 1). Considering Early Sporogonic Stages (ESS) counts, i.e. numbers of zygotes, retort forms and ookinetes, none of the 3 tested compounds resulted different from DMSO controls, whereas a slight effect was observed with DHA and AS on ookinete development (OD). Specific counts of retort forms and elongated ookinetes were reduced by about 50% in wells incubated with DHA and AS at 75μM compared to controls. Testing DHA and AS at lower concentrations (50 and 10 μM) confirmed AS inhibitory activity on ookinete maturation. In comparison, the TB reference product NA at 10 μM inhibited ESS and OD by about 45 and 56% respectively (Table 1).

**Table 1:** Effect of artemisinin derivatives on the development of *P.berghei* early sporogonic stages in the ookinete development assay (ODA).

Compound	Concentration (μM)	% Inhibition [CI_95%_]	
		ESS^a^	OD
ART^b^	75	0.6 [0.0-0.7]	17.1 [16.0-18.3]
	75	5.3 [3.9-6.4]	53.3 [52.1-54.5]
DHA	50	16.9 [15.5-18.4]	32.6 [31.1-34.2]
	10	12.5 [11.3-13.7]	28.7 [27.6-29.8]
	75	1.7 [0.9-2.4]	50.0 [48.4-51.0]
AS	50	26.1 [24.7-26.5]	66.1 [65.0-67.0]
	10	5.2 [4.6-5.9]	40.0 [39.4-51.6]
NA	10 **(**μg/ml)	45.0 [44.5-45.3]	55.8 [54.4-57.0]

^a^ ESS = Early Sporogonic Stages; OD = Ookinete Development.

^b^ ART = Artemisinin; DHA **=** Dihydroartemisinin; AS **=** Artesunate; NA **=** NeemAzal®

### 3.2 Artemisinin and artesunate TB characteristics in the *P. berghei* Direct Feeding Assay

**Figure 1:**
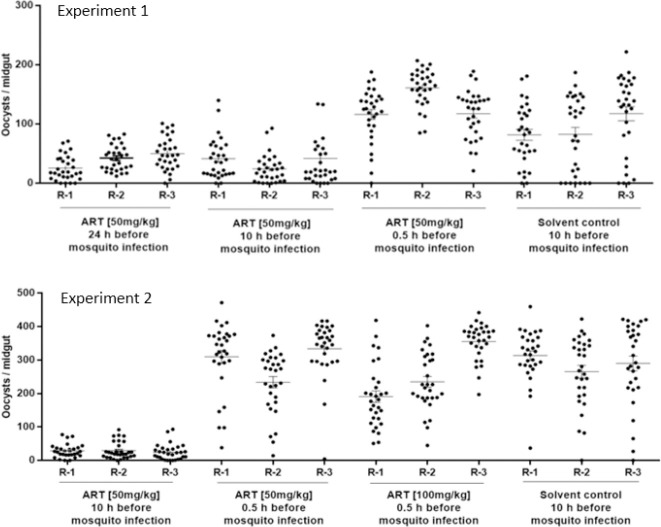
Transmission blocking activity of ART given at various dosages and time points before mosquito infection. Two consecutive experiments were performed to explore the *in-vivo* transmission blocking activity of ART given to gametocytemic mice (i.p.) at various doses and time points before mosquito infection. In experiment-1, gametocytemic mice were treated with ART at 50 mg/kg at 24, 10 and 0.5 hrs before mosquito infection. Whereas, experiment-2 was conducted to assess the TB activity of ART given at 50 and 100mg/kg 10 h and 0.5h prior to mosquito infection. Each treatment group was done in triplicate mouse/mosquito cages (replicates: R-1, R-2, and R-3).

Experiments in the DFA were performed to explore gametocytocidal characteristics of artemisinins in the murine model. ART i.p. administrated to gametocytemic mice at 50 mg/ kg on day 3 after mouse infection and 24 h before mosquito infection, reduced oocyst density by about 60% (Table 2 and Figure 1), suggesting activity on young *P. berghei* gametocytes, present in the mouse plasma at this time point of administration. The same dose given 3.5 days after mouse infection (presence of mature gametocytes) decreased oocyst density by 75 % evidencing activity also on mature sexual parasite stages in the rodent host. Impact observed on oocyst density was not accompanied by a reduction of prevalence of infection (Table 2). No impact was found after ART administration 30 min before mosquito infection neither with the 50 nor 100 mg/kg dose. This result indicates no effects on mature gametocytes at this relatively short exposure time.

**Table 2:** ART transmission blocking effects according to dose and time of administration to gametocytemic mice before mosquito infection.

Treatment mg/kg	Exp^a^	Days after mouse infection^b^	Hours before mosquito infection	Geom. mean oocyst density^c^ [CI_95%_]	Percent oocyst reduction [CI_95%_]	Prevalence of infection % [CI_95%_]
Control^d^	1	3.5	10	88.6 [67.0-110.2]	-	87.0 [65.1-108.3]
	2	4	0.5	272.5 [236.5-308.4]	-	97.8 [61.8-133.7]
ART	1	3	24	34.7 [26.4-43.0]	60.9 [52.6-69.2]	94.0 [86.1-102.7]
50 mg/kg	2	3.5	10	67.0 [51.1-82.9]	75.4 [59.6-91.3]	96.7 [80.8-112.5]
ART	1	4	0.5	124.9 [111.9-139.0]	5.2 [0-38.5]	97.8 [84.8-112.9]
50 mg/kg	2	4	0.5	258.3 [225.0-291.7]	5.4 [0-35.5]	100 [66.7-133.3]
ART 100 mg/kg	2	4	0.5	245.1 [225.1-292.3]	10.0 [0-38.1]	100 [71.9-128.0]

^a^ Exp. = Experiment number.

^b^ In *P. berghei* gametocytes can be morphologically distinguished on Giemsa slides on day 3 after acyclic mouse infection. Full sized, infective gametocytes appear on day 4, thus treatment on day 3 and 3.5 targets young and mature gametocytes, respectively.

^c^ Oocyst density was calculated considering infected mosquitoes only.

^d^ Control: Mice treated with distilled water containing 10% DMSO and 10% Tween80.

**Figure 2:**
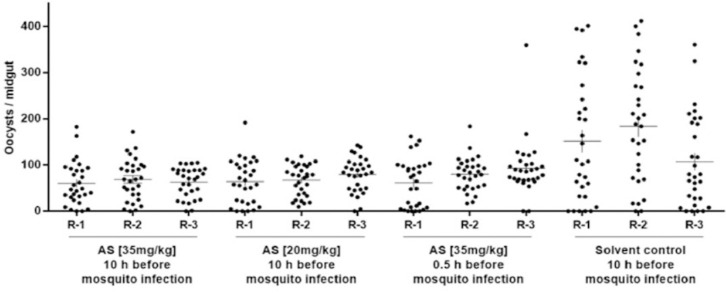
Transmission blocking activity of artesunate given at various dosages and time points before mosquito infection. AS was given to gametocytemic mice at 20 and 35 mg/kg 10 hrs before mosquito feeding. Each treatment group was done in triplicate mouse/mosquito cages (replicates: R-1, R-2, and R-3).

AS, administrated at 35 mg/kg and 20 mg/kg to gametocytemic mice 3.5 days after mouse infection, reduced at both dosages oocyst densities by about 50% (Table 3 and Figure 2), evidencing capacity to interfere with mature *P. berghei* gametocytes present in the mouse plasma at this time point. Unlike ART, AS administrated 30 min before mosquito feeding (at 35 mg/kg) was able to reduce oocyst abundance to about half of that of controls, that might reflect activity on mature gametocytes even at short exposure and/or on early sporogonic stages. As in the case of ART, impact observed on oocyst density was not accompanied by a reduction of prevalence of infection (Table 3, Figure 2).

**Table 3:** Artesunate transmission blocking effects according to dose and time of administration to gametocytemic mice before mosquito infection.

Treatment (mg/kg)	Days after mouse infection^a^	Hours before mosquito infection	Geom. mean Oocyst density^b^ [CI_95%_]	Percent oocyst reduction [CI_95%_]	Prevalence of infection % [CI_95%_]
Control^c^	3.5	10	120.3 [77.6-162.9]	-	87.8 [45.1-130.5]
AS	3.5	10	49.7 [34.9-64.4]	58.7 [43.9-73.2]	95.5 [81.4-119.7]
35 mg/kg	4	0.5	71.6 [50.6-86.1]	43.2 [37.2-65.1]	93.3 [75.5-111.1]
AS 20 mg/kg	3.5	10	59.1 [45.0-73.3]	50.8 [24.7-61.2]	96.0 [80.8-110.3]

^a^ In *P. berghei* gametocytes can be morphologically distinguished on Giemsa slides on day 3 after acyclic mouse infection. Full sized, infective gametocytes appear on day 4, thus treatment on day 3 and 3.5 targets young and mature gametocytes, respectively.

^b^ Oocyst densities (geometric mean oocysts/midgut) were considered only on oocyst positive mosquitoes.

**^c^** Control: Mice treated with 5% NaHCO_3_ in 0.85% normal saline.

Having confirmed activity of ART and AS on mature gametocytes of *P. berghei* (3.5 days after mouse infection), 10 h before mosquito feeding was chosen as time point for ART and AS administration in the 2TB-DFA.

### 3.3 Transmission blocking IC_50_ interval estimates for artemisinin, artesunate and NeemAzal®

In order to be able to appreciate cooperative effects of two TB compounds in the 2TB-DFA, compounds needed to be delivered at sub-optimal TB doses. Such doses were assessed for AS, ART and NA by a series of dose range experiments that allowed to estimate IC_50_ intervals for each compound / product.

**Figure 3:**
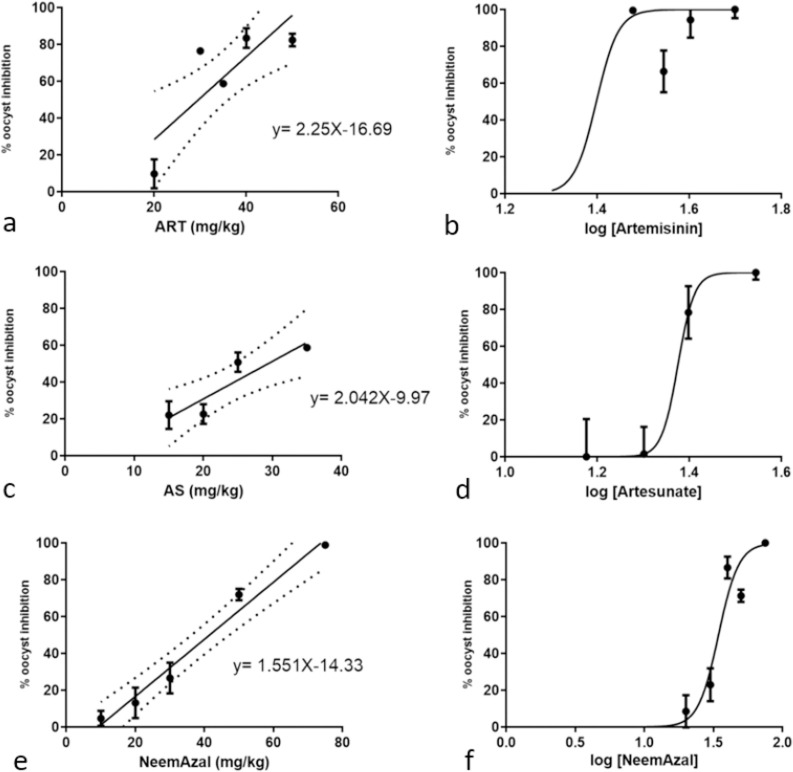
Dose response characterisation of artemisinin, artesunate and NeemAzal® by linear regression (a, c, e) and calculation of IC_50_ and CI_95_ values based on log transformed dose data and nonlinear regression (b, d, f); artemisinin (a, b): goodness-of-fit R-squared = 0.63; IC_50_ = 25mg/kg (CI_95_: 16 - 30); artesunate (c, d): goodness-of-fit R squared = 0.69; IC_50_ = 20mg/kg (CI_95_: 14 - 28); NeemAzal® (at aza-

For NA, tested at an azadirachtin A doses of 75, 50, 40, 30, 20 and 10 mg/kg (NA contains azadirachtin A at about 50%), a 50% reduction of oocyst density was observed in the dose range of 31 to 38 mg/kg [IC_50_ = 35 mg/kg; CI_95%_: 31-38] (Figure 3). The half maximal inhibitory dose of ART was estimated to range between 16 and 30 mg/kg [IC_50_ = 25mg/kg; CI_95%_: 16-30], based on experiments performed at 50, 40, 35, 30 and 20 mg/kg. AS tested at 40, 35, 25, 20 and 15mg/kg, showed an IC_50_ interval ranging from 14 to 28 mg/kg [IC_50_ = 20mg/kg; CI_95%_: 14-28]. Correlation of drug doses and oocyst densities assessed by linear regression yielded the following goodness-of-fit R-Squared values for ART: R= 0.63, AS: R = 0.69 and NA: R=0.93 (Figure 3).

### 3.4 TB effects of artemisinin and artesunate in combination with NeemAzal® in the 2TB-DFA

In each 2TB-DFA four treatment groups were included: 1. Combination treatment group: Gametocytemic mice treated with ART or AS 10 hrs before mosquito feeding and with NA 1h before feeding, giving both compounds at a dose ranging in the estimated IC_50_ interval; Group 2. and 3. Single compound controls: Mice treated with ART or AS at 10 h or NA 1 h before mosquito feeding at the same dosages as in the combination treatment group; 4. solvent controls.

When ART was administered to gametocytemic mice at 35 mg/kg in combination with NA at 40 mg/kg dissection of mosquito midguts on day 6 after mosquito infection revealed a reduction in oocyst density of 94%; mean oocyst counts of 12 were registered in the combination group compared to 204 in solvent control mosquitoes (Table 4, experiment 1). The single compound treatment with ART-35 reduced oocyst density by 42% and that of NA-40 by 86%. Applying ‘Highest Single Agent’ analysis, no indication on a cooperative effect emerges comparing the reduction of oocyst density obtained by the ART-35 + NA-40 combination with that of NA, the highest single agent (Combination Index CI=0.915; p=0.306). However, a positive effect resulted when comparing the combination treatment with ART-35 single compound treatment (CI=0.447; p<0.0001), indicating a beneficial effect of NA post-treatment after ART administration. Considering prevalence as parameter to compare treatment effects, the two compounds in combination were significantly more effective than both individual treatments, i.e. ART-35 (CI=0; p<0.0001) and NA-40 (highest single agent CI=0.339; p=0.0024). Given the almost complete blockage of transmission with ART-35+NA-40 combination, the subsequent experiment was performed with ART at 25 mg/kg and NA at 35 mg/kg. Mean oocysts numbers observed in the combination group amounted to 5.8 compared to 125 in the solvent group, corresponding still to a reduction of 95.4% (Table 4, experiment 2). The single compound treatments with ART-25 and NA-35 reduced oocyst abundance by 67% and 66%, respectively. In this experiment, ‘Highest Single Agent’ analysis, comparing ART-25 + NA-35 combination with ART-25, the ‘highest single agent’ in this experiment, indicated a cooperative effect of the combination, considering the parameter oocyst density (CI=0.737; p=0.0091) as well as prevalence of infection (CI=0.235; p=0.0142). Comparing the combination treatment with the single compound NA-35 a positive effect of the combination was found considering oocyst density (CI=0.695; p=0.0042) but not prevalence of infection (CI=0.529; p=0.1612; Table 4).

**Table 4:** Transmission blocking activity of Artemisinin (ART) and NeemAzal® (NA) single and combination treatment in the 2TB-DFA.

	Treatment mg/kg, (hrs before mosquito feeding)	Replicate	Oocyst density by replicate ^a^ [CI_95%_]	Prevalence of infection [infected/total examined]	Oocyst density by treatment ^a^ [CI_95%_]	% reduction of oocyst density by treatment [CI_95%_]	Prevalence of infection by treatment [CI_95%_]	% reduction of prevalence by treatment [CI_95%_]
Experiment 1	ART 35	1	178.0 [149.2-206.7]	100 [30/30]	119.5	41.5	96.7	
	mg/kg	2	121.3 [90.2-152.4]	100 [30/30]	[92.0-147.0]	[14.3-69.0]	[69.2-124.2]	0
	(10)	3	59.1 [36.6-81.7]	90.0 [27/30]				
							
	NA 40	1	48.5 [28.2-68.9]	83.3 [25/30]	38.1	86.3	74.4	20.3
	mg/kg	2	26.1[9.4-42.8]	86.7 [26/30]	[14.4-41.4]	[72.8-99.8]	[60.9-87.9]	[0-43.2]
	(1)	3	9.2 [5.7-12.6]	53.3 [16/30]				
							
	ART 35	1	12.4 [6.5-18.3]	43.3 [13/30]	11.7	94.3	37.7	59.6
	(10) +	2	13.9 [10.4-17.3]	33.3 [10/30]	[7.8-15.7]	[90.3-98.2]	[33.9-41.7]	[53.4-66.1]
	NA 40 (1)	3	8.9 [6.5-11.4]	36.7 [11/30]				
	Solvent	1	185.2 [150.4-220.0]	93.3 [28/30]	204.3		93.3	
	control ^b^	2	197.8 [158.5-237.2]	86.7 [26/30]	[171.0-237.6]	-	[60.0-126.6]	-
	(10)	3	229.9 [204.1-255.6]	100 [30/30]				
Experiment 2	ART 25	1	33.8 [4.3-47.8]	90.0 [27/30]	38.1	69.6	92.2	7.8
	mg/kg	2	28.9 [12.9-44.9]	90.0 [27/30]	[16.6-65.9]	[48.2-91.1]	[70.8-113.7]	[7.2-11.4]
	(10)	3	51.6 [34.1-61.0]	93.3 [28/30]				
							
	NA 35	1	24.8 [10.7-43.2]	76.7 [23/30]	42.6	66.0	82.2	17.8
	mg/kg	2	28.4 [8.8-57.8]	86.7 [26/30]	[9.4-64.4]	[45.8-86.2]	[62.0-102.4]	[12.3-22.8]
	(10)	3	74.7 [47.9-92.2]	83.3 [25/30]				
							
	ART 25	1	6.9 [3.0-11.9]	73.3 [22/30]	5.8	95.4	66.4	33.6
	(10) +	2	6.5 [3.6-9.5]	73.3 [22/30]	[3.4-10.2]	[92.1-98.7]	[63.3-69.9]	[21.1-47.3]
	NA 35 (1)	3	4.0 [0.8-9.1]	53.3 [16/30]				
	Solvent	1	109.4 [78.5-140.2]	100 [30/30]	125.4		100	
	Control ^b^	2	123.6 [93.6-153.7]	100 [30/30]	[94.5-156.3]	-	[69.1-130.9]	-
	(10)	3	143.3 [111.5-175.1]	100 [30/30]				

^a^ Oocyst densities (geometric mean oocysts/ midgut) were considered only on oocyst positive mosquitoes. ^b^ Solvent control: mice treated with PBS (pH 6.5) containing 5% ethanol, 10% DMSO and 7.5% Tween80.

Mosquitoes fed on gametocytemic mice treated with the combination of AS at 20mg/kg and NA at 35 mg/kg, reduced oocyst density by 71% compared to the solvent control (Table 5). In this experiment, none of the treatments affected prevalence of mosquito infection. The mean number of oocysts enumerated in the AS-20 + NA-35 group amounted 35.6 (two replicates only, one mouse died at treatment), that of control mosquitoes fed on solvent-treated mice 121 and that of AS-20 and NA-35 single compound treatment 70 and 79, respectively. ‘Highest Single agent’ analysis indicates a cooperative effect of the combination treatment compared with either, the AS-20 (CI=0.591; p=0.0247) and the NA-35 (CI=0.493; p=0.0056) single compound treatment.

**Table 5:** Transmission blocking activity of artesunate (AS) and NeemAzal® (NA) single and combination treatment in the 2TB-DFA.

Treatment mg/kg (hrs before mosquito feeding)	Replicate	Oocyst density by replicate ^b^ [CI_95%_]	Prevalence of infection [infected/total examined]	Oocyst density by treatment [CI_95%_]	% reduction of oocyst density by treatment [CI_95%_]	Prevalence of infection by treatment [CI_95%_]	% reduction of prevalence by treatment [CI_95%_]
AS 20 mg/kg (10)	1	80.6 [55.4-105.8]	100 [30/30]				
	2	92.6 [70.2-115.0]	93.3 [28/30]	70.2	41.9	92.2	7.8
	3	37.2 [14.7-59.7]	83.3 [25/30]	[46.8-93.5]	[18.5-65.2]	[68.9-115.6]	[0-17.1]
NA 35 mg/kg (1)	1	65.6 [20.8-110.3]	93.3 [28/30]				
	2	64.7 [29.3-100.1]	76.7 [23/30]	79.0	34.5	88.9	10.1
	3	106.8 [76.5-137.1]	96.7[29/30]	[42.2-115.8]	[2.3-71.3]	[52.1-125.7]	[0–21.4]
AS 20 mg/kg (10) **+** NA 35 mg/kg (1)	1	54.5 [37.4-72.0]	100 [30/30]				
	2	16.8 [^a^10.9-22.7]	100 [30/30]	35.6	70.5	100	
	3			[24.2-47.1]	[59.0-81.9]	[85.9-111.5]	0
Solvent control^c^ (10)	1	107.4 [74.1-140.8]	100 [30/30]				
	2	157.8 [124.1-191.4]	100 [30/30]	120.7		98.9	
	3	96.8 [68.1-125.6]	96.7 [29/30]	[88.7-152.6]	-	[66.9-130.8]	-

^a^ Mouse died shortly after NA administration. **^b^** Oocyst densities (geometric mean oocysts/ midgut) were considered only on oocyst positive mosquitoes. **^c^** Solvent control: mice treated with normal saline containing 5% ethanol, 10% DMSO and 7.5% Tween80.

## 4 Discussion

We here propose a murine malaria assay designed to estimate additional benefits of transmission blocking (TB) drugs when given in combination. The study was motivated by the limited gametocytocidal activity of ACTs currently employed to cure uncomplicated malaria and the need to cope with persistent gametocyte circulation in individuals after ACT treatment, which constitute infective reservoirs to mosquito vectors [3,27]. The here developed double TB-drugs Direct Feeding Assay (2TB-DFA) uses *P. berghei* as malaria parasite, BALB/c mice and *An. stephensi* mosquitoes as vertebrate and invertebrate host, respectively. Employment of sub-optimal TB dosages (ranging within the IC_50_ interval) of artemisinin (ART), artesunate (AS) and a secondary TB compound enabled the 2TB-DFA to discern cooperative effects of the compounds when given in combination. As a secondary (post-treatment) TB agent NeemAzal® (NA), an azadirachtin A rich product, known to affect sporogonic development, was used. Groups of gametocytemic mice were treated on day 4 after mouse infection with ART, AS or NA alone or in combination (ART + NA or AS + NA), measuring as assay read out mosquito oocyst prevalence and density. When administrating ART or AS to *P. berghei* gametocytemic mice 10 h before mosquito feeds and adding NA as post-treatment 1h before mosquito feeds, the 2TB-DFA was able to discern cooperative effects of the combination treatments evidenced by ‘Highest Single Agent’ analysis [25,26].

A first series of experiments was performed to characterise the parasite stage specific transmission blocking effects of ART and AS in the mouse model, given the substantially different process of gametocytogenesis in the human parasite *P. falciparum* compared to the rodent *P. berghei* species. In *P. falciparum*, malaria development of male and female gametocytes requires 7 to 15 days [27-29] whereas *P. berghei* sexual forms are developed in 22-28 h and persist for another 1-2 days in circulation [30]. Mice infected by acyclic passage, exhibit developing gametocytes on day three (morphologically distinguishable but not full sized) after infection and mature infective forms on day four. In our experiments, ART administrated (i.p.) to gametocytemic mice at 50 mg/kg on day 3 after mouse infection, reduced oocyst density by about 60%, revealing activity on young gametocytes. The same dose given 3.5 days after mouse infection, i.e. at completion of the sexual reproduction, decreased oocyst density by 75 %, evidencing activity also on mature sexual stages. Similarly, AS was found to interfere with mature *P. berghei* gametocytes *in vivo*, reducing oocyst density by 50% after administration at 35 mg/kg to mice on 3.5 days after mouse infection. Gametocytocidal activity of ART ad AS against *P. falciparum* is well established and has been characterised by various *in vitro* studies [9]. For instance, in screens performed by Lucantoni *et al.* [31], ART and AS inhibited 50% of stage I-III *P. falciparum* gametocyte development at 12.9 and 2.9 nm, respectively and mature stage V gametocytes at 25.9 and 44.9 nm. Thus, although gametocytogenesis is distinct in *P. berghei* and *P. falciparum*, both parasite species are susceptible to the gametocytocidal action of ART and AS, suggesting the *P. berghei in vivo* DFA to be a valid proxy for the human malaria parasite to explore transmission blocking beneficial effects of TB compounds given as post-treatment after artemisinin derivatives administration.

In further characterisation studies ART, dihydroartemisinin (DHA) and AS were found to interfere insignificantly with *P. berghei* early sporogonic development *in vitro*. Testing the compounds at a primary concentration of 75μM in the ookinete development assay (ODA), total counts of early sporogonic stages (zygotes, retort forms, ookinetes) were similar in treated and solvent control wells. The read out ‘total early sporogonic stages’ informs about compound activity on biological processes upstream of zygote formation, i.e. gamete formation and fecundation, meaning for ART and AS no activity on pre-zygote formation processes. Differential counts of retort forms and elongated ookinetes at 75, 50 and 10 μM test concentrations, revealed 50% reduction of these post-zygote forms by AS when tested at 10 μM, indicating effects of the compound on processes of ookinete formation. For comparison, Delves *et al.* [10] reported about 45% inhibition of early sporogonic stage development (all stages) for ART and 40% for AS, screening compounds at 10 μM in the *P. berghei* ODA. Experiments performed in the Standard Membrane Feeding Assay (SMFA) by the same group, confirmed minor effects of DHA and AS on sporogonic stages of *P. berghei* in the mosquito; feeds of gametocytemic mouse blood with DHA and AS at 10 μM reduced oocyst densities by 40 and 20%, respectively, ART did not have any impact on mosquito infection [10]. In more recent SMFA experiments with mosquito feeds on *P. falciparum* gametocyte cultures, Bolscher *et al.* [32] found a reduction of oocyst densities of about 30% by DHA at 1 μM and of about 20% by AS at the same concentration, evidencing a slightly stronger effect of the compounds on *P. falciparum* than on *P. berghei* [32]. Overall, these results suggest some effects of artemisinin derivatives on sporogonic development of both *P. berghei* and *P. falciparum*, albeit in terms of activity far away from the levels observed against gametocytes.

In our *in vivo* DFA experiments we found a reduction in oocyst density (about 43%) with AS when treating gametocytemic mice (i.p. 35 mg/kg) just 30 min before mosquito infection. Given the half live of artemisinins in mice of about 25 min [33], the impact on mosquito infection might have been due to AS gametocytocidal activity, but effects on early sporogonic stages of remaining dihydroartemisinin in the mouse plasma cannot be ruled out. For clarifying this point, a future DFA experiment should include pharmacokinetic evaluation.

Since the objective of this study was to setup a DFA assay capable to estimate beneficial effects of a TB compound given after an ACT course, we opted to administrate artemisinin derivatives at 10 h before mosquito infection in the 2TB-DFA. Given at this time point, the assay measures TB effects through artemisinins activity on gametocytes only and the effects of a secondary treatment given 1 h before mosquito feeding is not influenced by drug interactions of the 2 tested compounds.

Dose range experiments with ART, AS and NA, aimed at assessing IC_50_ values of the 3 compounds and of the NA product in the DFA, revealed considerable variability of compounds’ TB efficacy from one experiment to another. This is not surprising, considering a double *in vivo* situation where numerous mouse factors influence gametocyte density and infectivity and various mosquito factors affect sporogonic development. Thus, IC_50_ estimates reported here are given as ranges corresponding to the 95% confidence intervals and amount for ART: 16–30 mg/kg, AS: 14-28 mg/kg and for NA (calculated on its azadirachtin A content): 31-38 mg/kg. In the 2TB-DFA experiments, dosages of ART, AS and NA ranging within the 95% confidence interval were used.

The established 2TB-DFA protocol involves 4 treatment groups, namely combination treatment (group 1), single compound controls (group 2 and 3) and solvent control (group 4). Overall, the 3 experiments conducted, 2 with ART + NA and one with AS + NA, allowed to discern cooperative effects of the combination treatments compared to the single compound treatments. ART given at 35 mg/kg (10 h before mosquito infection) plus NA at 40 mg/kg (1h before mosquito infection) reduced oocyst density by 94% and prevalence of infection by 59%. The single compound treatment with ART-35 yielded a 42% reduction of oocyst density (no effect on prevalence) and with NA-40 an 86% reduction of oocyst density and 20% of prevalence was observed. Applying ‘Highest Single Agent’ analysis [25,26], a significant cooperative effect emerged considering prevalence as readout parameter in the comparison of the combination effect with that of the ‘Highest Single Agent’, which was NA-40 in this experiment (Combination Index CI=0.339; p=0.0024). Considering oocyst density as readout, no positive effect resulted from the comparison of the combination treatment with NA-40 single compound treatment. However, this was the case with ART-35 (CI=0.447; p<0.0001), indicating a beneficial effect of NA-40 post-treatment after ART administration. Analysis of the second 2TB-DFA experiment, in which the dosage of ART was reduced to 25 mg/kg and that of NA to 35mg/kg, yielded a consistent picture of ‘cooperation’ considering the parameter oocyst density. A significant cooperative effect of the ART-25 + NA-35 combination emerged from the comparison with either of the single agent treatments on oocyst density (ART-25: CI=0.737 p=0.0091; NA-35: CI=0.695 p=0.0042). Similarly, experiments with AS-20 and NA-35, showed a cooperative effect of the combination compared to both single agents when looking at oocyst densities (AS-20:CI=0.591 p=0.0247; NA-35: CI=0.493 p=0.0056). Hence, in this set of experiments, oocyst density emerged to be more appropriate for discerning cooperative effects than prevalence of infection.

In summary, the 2TB-DFA, supported by ‘Highest Single Agent’ analysis appears suitable for the profiling of new TB candidates which could substitute primaquine as a post-treatment to ACT courses. Reviewing the literature, we did not come across other *in vivo* protocols addressing the issue of TB combination treatment. A few studies dealt with combinations of artemisinins with herbal preparations targeting asexual blood stages. Sanella *et al.* [34] investigated the combination of AS at 10 mg/kg (sub-optimal dosage) with a leaf decoction of *Carica papaya* in a 14 days suppressive test and were able to evidence an increased impact on parasitemia in the combination treated mice compared to single treatment AS and papaya controls [34]. Somsak *et al.* [35] employed the Peter’s four day protocol to test aqueous crude extracts of *Gynostemma pentaphyllum* or *Moringa oleifera* leaves in combination with artesunate (suboptimal dosages) and found an additional decrease of parasitemia in the extract-artesunate combination group compared with the single AS or the extract treatment [35]. Such studies may prepare the ground to ask whether combining ACTs with antimalarial herbal preparations for malaria management can delay the emergence and diffusion of *P. falciparum* parasite strains resistant to artemisinins. Without doubt, combination candidates (compounds or plant preparations) that act also on gametocytes and sporogonic stages targeting the small ‘bottleneck’ parasite populations [36]**,** would be more effective for this purpose. A recent Ghanaian study, showed 5 out of 10 commercial plant preparations to be active against *P. falciparum* asexual stages *in vitro* (IC_50_ < 5 μg/ml) and one, containing *Cryptolepis sanguinolenta* and *Azadirachta indica* able to inhibit survival of late stage gametocytes at 1 μg/ml [37].

## 5 Conclusions

In conclusion, we here presented a 2TB-DFA protocol suitable for profiling TB drug combinations designed to reduce malaria transmission but which may also be able to cope with drug resistance. We hope that this work may stimulate other parasitology labs to explore, *in vivo*, the potential benefits of combining drugs and phytomedicines with various chemical, pharmacological and transmission blocking characteristics. Numerous *in vitro* characterised gametocytocidal compounds of natural and synthetic origin as well as plant extracts are on the bench.
